# Chromosomal Analysis in *Crotophaga ani* (Aves, Cuculiformes) Reveals Extensive Genomic Reorganization and an Unusual Z-Autosome Robertsonian Translocation

**DOI:** 10.3390/cells10010004

**Published:** 2020-12-22

**Authors:** Rafael Kretschmer, Ricardo José Gunski, Analía del Valle Garnero, Thales Renato Ochotorena de Freitas, Gustavo Akira Toma, Marcelo de Bello Cioffi, Edivaldo Herculano Corrêa de Oliveira, Rebecca E. O’Connor, Darren K. Griffin

**Affiliations:** 1 School of Biosciences, University of Kent, Canterbury CT2 7NJ, UK; rebeckyoc@gmail.com (R.E.O.); d.k.griffin@kent.ac.uk (D.K.G.); 2 Laboratório de Citogenética e Evolução, Departamento de Genética, Instituto de Biociências, Universidade Federal do Rio Grande do Sul, Porto Alegre 91509-900, Rio Grande do Sul, Brazil; thales.freitas@ufrgs.br; 3 Laboratório de Diversidade Genética Animal, Universidade Federal do Pampa, São Gabriel 97300-162, Rio Grande do Sul, Brazil; ricardogunski@unipampa.edu.br (R.J.G.); analiagarnero@unipampa.edu.br (A.d.V.G.); 4 Laboratório de Citogenética de Peixes, Departamento de Genética e Evolução, Centro de Ciências Biológicas e da Saúde, Universidade Federal de São Carlos, São Carlos 13565-905, São Paulo, Brazil; gustavo_toma@hotmail.com (G.A.T.); mbcioffi@ufscar.br (M.d.B.C.); 5 Laboratório de Cultura de Tecidos e Citogenética, SAMAM, Instituto Evandro Chagas, Ananindeua 67030-000, Pará, Brazil; ehco@ufpa.br; 6 Instituto de Ciências Exatas e Naturais, Universidade Federal do Pará, Belém 66075-110, Pará, Brazil

**Keywords:** birds, genome evolution, sex chromosomes, chromosomal rearrangements

## Abstract

Although cytogenetics studies in cuckoos (Aves, Cuculiformes) have demonstrated an interesting karyotype variation, such as variations in the chromosome morphology and diploid number, their chromosome organization and evolution, and relation with other birds are poorly understood. Hence, we combined conventional and molecular cytogenetic approaches to investigate chromosome homologies between chicken and the smooth-billed ani (*Crotophaga ani*). Our results demonstrate extensive chromosome reorganization in *C*. *ani*, with interchromosomal rearrangements involving macro and microchromosomes. Intrachromosomal rearrangements were observed in some macrochromosomes, including the Z chromosome. The most evolutionary notable finding was a Robertsonian translocation between the microchromosome 17 and the Z chromosome, a rare event in birds. Additionally, the simple short repeats (SSRs) tested here were preferentially accumulated in the microchromosomes and in the Z and W chromosomes, showing no relationship with the constitutive heterochromatin regions, except in the W chromosome. Taken together, our results suggest that the avian sex chromosome is more complex than previously postulated and revealed the role of microchromosomes in the avian sex chromosome evolution, especially cuckoos.

## 1. Introduction

In birds, males and females represent the homogametic (ZZ) and the heterogametic (ZW) sex, respectively [1,2,3]. Although the Z chromosomes are similar in size across all bird species, several intrachromosomal rearrangements have been observed among them [4]. On the other hand, the W chromosomes show great variability in sizes, morphologies, and gene content [2,3]. Typically, the Z chromosome is comparable in size with the fourth or the fifth chromosome pair, while the W is considerably smaller [1]. Nevertheless, chromosome expansion on both sex chromosomes can be observed in some lineages, such as in the Z chromosome of Piciformes [5,6] and in the W chromosome in some Caprimulgiformes [7], Gruiformes [8], and Psittaciformes species [9].

Interchromosomal rearrangements between autosomes and sex chromosomes are considered rare events among birds [2,3]. However, rearrangements between autosomes and sex chromosomes have been described for several mammalian orders, such as in Artiodactyla [10], Primates [11], and Rodentia [12]. To the best of our knowledge, there are only a few cases of this type of rearrangement in birds. Two different Z-autosome translocations were identified in white leghorn cockerels (chicken, *Gallus gallus*) and represent chromosomal abnormalities [13,14]. Telloni et al. [14] identified a reciprocal exchange between the Z and a microchromosome, and Zartman [13] described a reciprocal exchange between the Z and the long arms of chromosome one. Recently, a series of studies based on whole-genome data proposed complex trajectories of the sex chromosome in Sylvioidea (Passeriformes), where four independent autosome–sex chromosome fusions involving the Z, GGA4p, and segments from GGA3 and GGA5 have been identified [15,16,17,18]. Concerning W-autosome translocations, the only known case was described in *Pygoscelis adeliae*, a Sphenisciformes species with a rare (among birds) multiple sex chromosome system ♂ Z1Z1Z2Z2/♀ Z1Z2W type based on conventional cytogenetics analyzes [19]. The authors suggested that a translocation between the W and an unidentified autosome gave rise to this multiple sex chromosome system present in this species [19].

In the last decades, whole chromosome painting data (WCP) has been used to investigate the chromosome evolution of both autosomes and sex chromosomes of birds and has provided important insights into the chromosome evolution of this group [3,20]. Using this approach, chromosome signatures were identified, representing useful characters both for phylogenetic considerations and deciphering the avian genome evolution [3,20,21,22]. However, most of these studies are restricted to chicken chromosomes 1–10, limiting our understanding to macrochromosomes [3,20]. Recently, bacterial artificial chromosome (BAC) probes have been used in avian cytogenetics studies, revealing inter and intrachromosomal rearrangements not previously observed after WCP analysis [23,24,25]. One of the main contributions of BACs probes was the ability to identify the exact microchromosome involved in the interchromosomal rearrangements. Up to now, most of the avian orders studied with this approach showed the conservation of the ancestral pattern of microchromosomes organization [25]. However, fusions events involving microchromosomes have been found in Falconiformes and Psittaciformes [22,23,24,25]. Considering that only 10 [25] out of 40 avian orders [26] were analysed with microchromosomes probes so far, the organization of these small elements remains largely unknown among birds.

Although at first glance most species show the typical avian karyotype, such as seen in chicken (2n = 78) [3,20,21], some degree of variation is found among cuckoos (order Cuculiformes). Cuckoos are cosmopolitan birds, widely distributed on all tropical and temperate continents, comprising only one family (Cuculidae) with 32 genera and 149 species [26]. The genus-level phylogeny of cuckoos based on mitochondrial DNA sequences divides them into five subfamilies: Crotophaginae and Neomorphinae in the New World, Centropodinae and Couinae in the Old World, and Cuculinae, which has mainly species in the Old World but includes a clade of New World cuckoos represented by *Coccycua*, *Piaya*, and *Coccyzus* [27].

Cytogenetics studies with cuckoos’ species are still scarce and based mostly on conventional cytogenetics approaches [28,29,30]. These studies demonstrated a variation in karyotype organizations with 2n ranging from 64 in *Crotophaga major* (Crotophaginae) [29] to 90 in *Piaya cayana* (Cuculinae) [30]. WCP experiments using *Gallus gallus* and *Leucopternis albicollis* macrochromosomes probes have been applied only to two species: *Guira guira* (Cuculinae) and *Piaya cayana* (30), revealing that the chromosome evolution in cuckoos involved mainly fissions and fusions events when compared with *G*. *gallus*. However, fusions were more common in *G*. *guira*, resulting in a low diploid number (2n = 76) in contrast with a high diploid number in *P*. *cayana* (2n = 90) [30]. Besides, some gaps in the macrochromosomes of both species were not hybridized by the *G*. *gallus* and *L*. *albicollis* probes, suggesting the occurrence of microchromosomes fusions [30].

To investigate the chromosomal changes during the diversification of cuckoos, we mapped chicken BACs for chromosomes 1-28 and the Z and W sex chromosomes and some repetitive DNAs sequence in the chromosomes of *Crotophaga ani* (Crotophaginae). Our results showed extensive genomic reorganization in this species, involving fissions and fusions of macrochromosomes and microchromosomes. The most unexpected finding was a Z-autosome Robertsonian translocation, which is a rare event among birds. Taken together, our results bring new insights into evolutionary trends within birds, especially cuckoos.

## 2. Material and Methods

### 2.1. Specimens and Chromosome Preparation

Two females’ specimens of *C*. *ani* were collected at Porto Vera Cruz, the Rio Grande do Sul State, Brazil, and one female was collected at Santa Barbara, the Pará State, Brazil, and analysed in this study. The individuals were captured using mist nets in their natural environment, following the protocols authorized by the System of Authorization and Information in Biodiversity (SISBIO, number 33860-1, 44173-1, and 68443-1) and the Ethics Committee on Animal Experimentation of Universidade Federal do Pampa (CEUA number 018/2014), and Universidade Federal do Pará (CEUA number 170/2013). Metaphase chromosome spreads were obtained from fibroblast cell cultures, established from skin biopsies, according to Sasaki et al. [31], with modifications. The cells were cultured in Dulbecco’s modified Eagle’s medium (DMEM) supplemented with 15% foetal bovine serum, 2% penicillin streptomycin, and 1% L-glutamine at 37 °C. The cell lines were cultured until the third passage and the diploid number was checked in each passage to ensure the maintenance of the original chromosome organization. Metaphase chromosome spreads followed standard protocols: treatment with colcemid (1 h), hypotonic solution (0.075 M KCl, 15 min), and fixation with 3:1 methanol/acetic acid.

### 2.2. Diploid Number, Karyotype Description, C-Banding, and G-Banding

The diploid number were identified by analysing at least 20 metaphase chromosome spreads per individual, conventionally stained with Giemsa 10% in 0.07 M phosphate buffer, at pH 6.8. Chromosomal morphology was determined according to Guerra [32]. The G-banding patterns of chromosomes were obtained using DAPI and propidium iodide [24]. The C-banding was performed according to Sumner [33].

### 2.3. Fluorescent in Situ Hybridization (FISH)

BAC probes from chicken (CH261) and zebra finch (TGMCBA) corresponding to GGA1-28 (except GGA16) and Z and W sex chromosomes were chosen and applied to metaphases of *C*. *ani* individuals collected at Porto Vera Cruz. The metaphases from the individual from Santa Barbara were not enough to perform this approach. Two BAC probes were selected for chromosomes GGA6-28, however, for the first five macrochromosomes (GGA1-5) and the Z chromosome, we applied more than two clones, to detect intrachromosomal rearrangements. Hence, 12 BAC clones were used for GGA1, 11 for GGA2, 8 for GGA3, 6 for GGA4, 4 for GGA5, and 3 for GGA Z. In total, 89 BAC clones were applied to *C*. *ani* metaphases (Table S1). The BAC clone isolation, amplification, labelling, and hybridization were performed following O’Connor et al. [25]. The chromosomes were counterstained with DAPI (blue), and the BAC probes were labelled with Texas Red (red) and FITC (green). The fluorescent in situ hybridization (FISH) results were confirmed by analysing at least 10 metaphase spreads per experiment.

Seven simple short repeats (SSRs; (CA)_15_, (CAC)_10_, (CAG)_10_, (CGG)_10_, (GA)_15_, (GAA)_10_ and (GAG)_10_), directly labelled with Cy3 during synthesis (Sigma, St. Louis, MO, USA), were mapped to metaphases of *C*. *ani*, according to Kubat et al. [34]. The SSRs mapping were performed to better characterize the chromosomal distribution of repetitive DNA sequences, especially in the Z and W sex chromosomes.

In order to identify the chromosomes bearing the nucleolus organizer regions, 18S rDNA fragments were obtained by polymerase chain reaction (PCR) as described by Cioffi et al. [35], labelled with Atto-488 by the nick translation kit, according to the manufacturer’s recommendations (Jena Bioscience, Jena, Germany), and mapped to *C*. *ani* metaphases.

### 2.4. Microscope Analysis and Image Capturing

Images of BAC FISH experiments were captured using a CCD camera and SmartCapture (Digital Scientific UK) system coupled on an Olympus BX61 epifluorescence microscope. Images of repetitive DNAs FISH experiments were captured using an Olympus BX50 microscope (Olympus Corporation, Ishikawa, Japan) with CoolSNAP. Final image processing was performed using Adobe Photoshop 7.0.

## 3. Results

### 3.1. Diploid Number, Karyotype Description, C-Banding, and G-Banding

**Figure 1:**
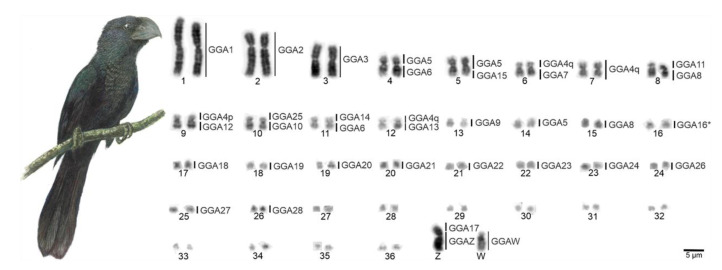
G-banded karyotype of *Crotophaga ani* female and homology maps with *Gallus gallus* (GGA, on the left). Scale bars = 5 µm.

**Figure 2:**
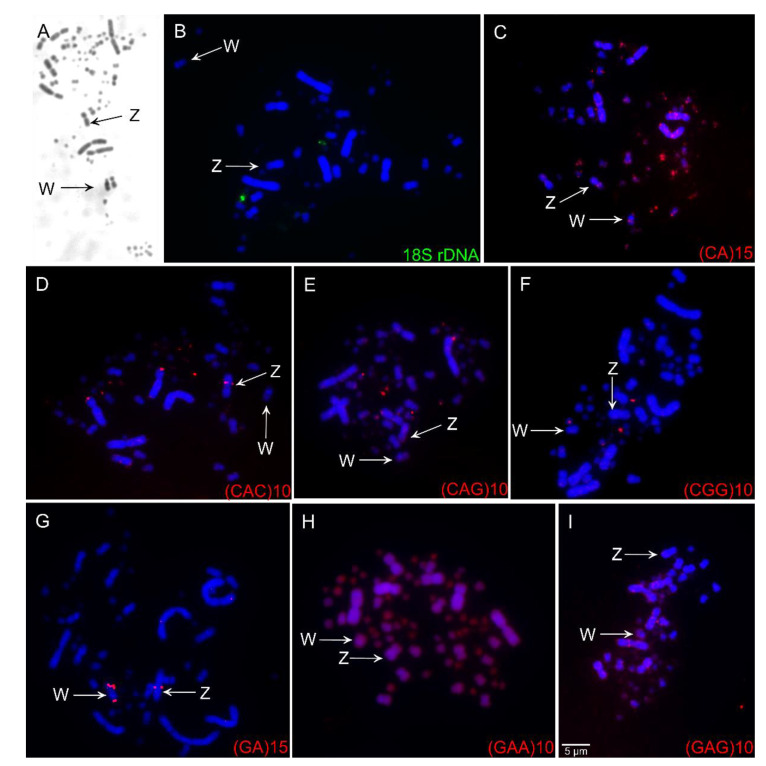
C-banding pattern (**A**), fluorescent *in situ* hybridization (FISH) experiment using 18S rDNA probes (**B**), and FISH experiments using simple short repeats (**C–I**) in *Crotophaga ani*. The chromosome probes used are indicated on the right bottom and the sex chromosomes (Z and W) are indicated by arrows. Scale bars = 5 µm.

The chromosome number of *C*. *ani* individuals was 74, with 14 pairs of macrochromosomes, including the Z and W chromosomes, and 23 microchromosome pairs (Figure 1). Pairs 1, 3, 4, 5, 7, 11, and 12 are metacentric, 2, 6, 9, and 10 are submetacentric, and 8 and 13 are acrocentric. The microchromosomes were considered telocentric. The Z and W are submetacentric and acrocentric, respectively. The constitutive heterochromatin was found only in the pericentromeric region of the W chromosome (Figure 2A).

### 3.2. Fluorescence in Situ Hybridization (FISH) of Repetitive Sequences

The 18S rDNA clusters were found in a single microchromosome pair (Figure 2B). All SSRs tested in our study produced reproducible signals (Figure 2C–I). In general, the SSRs sequences were preferentially accumulated in microchromosomes and in the Z and W chromosomes. The SSRs (CA)_15_ showed scattered signals in the first and second pairs, in several pairs of microchromosomes, in the pericentromeric region of chromosome Z and an interstitial region of the long arms of W. (CAC)_10_ hybridized in the terminal region of the short arms of chromosome 2, in an interstitial region in the long arms of Z, and four pairs of microchromosomes. (GAG)_10_ and (GAA)_10_ had scattered signals in all chromosomes. (GA)_15_ had signals only in the sex chromosomes—interstitial regions in the long arm of Z and W and the short arms of W. (CAG)_10_ had scattered signals in all chromosomes but a strong hybridization pattern in three pairs of microchromosomes. (CGG)_10_ hybridized only to two pairs of microchromosomes.

### 3.3. Fluorescence in Situ Hybridization (FISH) of Chicken and Zebra Finch BAC Clones

**Figure 3:**
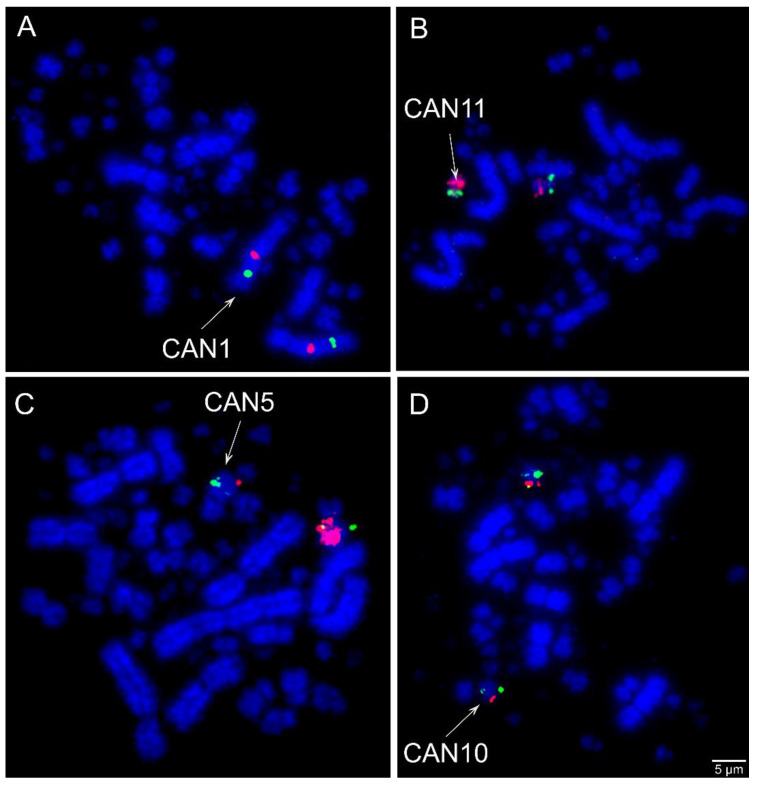
Examples of FISH experiments using chicken (CH261) and zebra finch (TGMCBA) bacterial artificial chromosome (BAC) probes in *Crotophaga ani*. (**A**) Chicken macrochromosome 1 CH261-118M1 (Red) and CH261-107E2 (green); (**B**) chicken microchromosome 14 CH261-122H1 (green) and chicken macrochromosome 6 CH261-49F3 (red) Texas Red; (**C**) chicken microchromosome 15 CH261-90P23 FITC and TGMCBA-266G23 (red); and (**D**) chicken microchromosome 25 CH261-59C21 (red) and chicken macrochromosome 10 CH261-71G18 (green). Scale bars = 5 µm.

**Figure 4:**
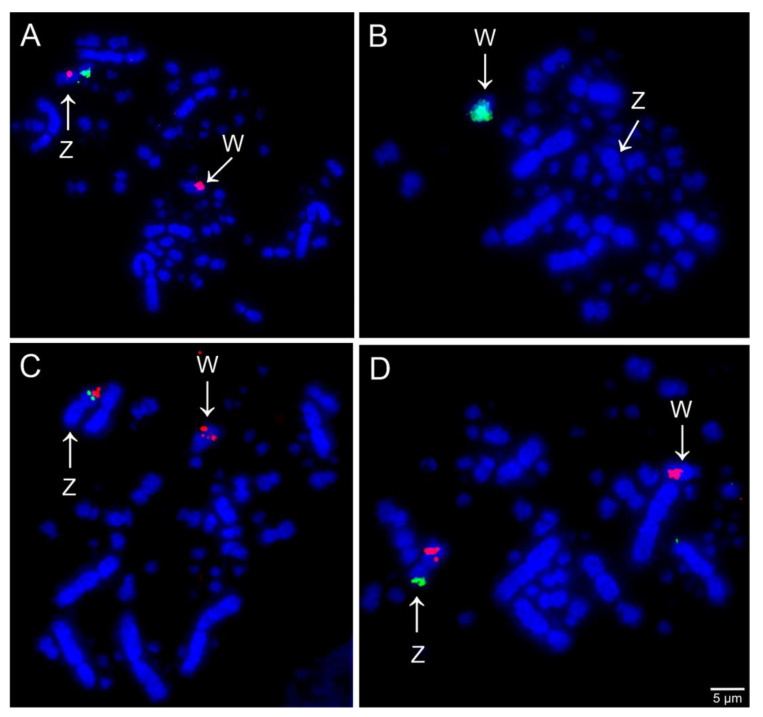
Examples of FISH experiments using chicken (CH261) and zebra finch (TGMCBA) BAC probes in *Crotophaga ani.* (**A**) Chicken chromosome Z CH261-129A16 (green) and CH261-133M4 (red); (**B**) chicken chromosome W CH261- 94E12 (green); (**C**) chicken microchromosome 17 TGMCBA-375I5 (green) and CH261-42P16 (red); and (**D**) chicken microchromosome 17 TGMCBA-375I5 (green) and chromosome Z CH261-133M4 (red). Scale bars = 5 µm.

**Figure 5:**
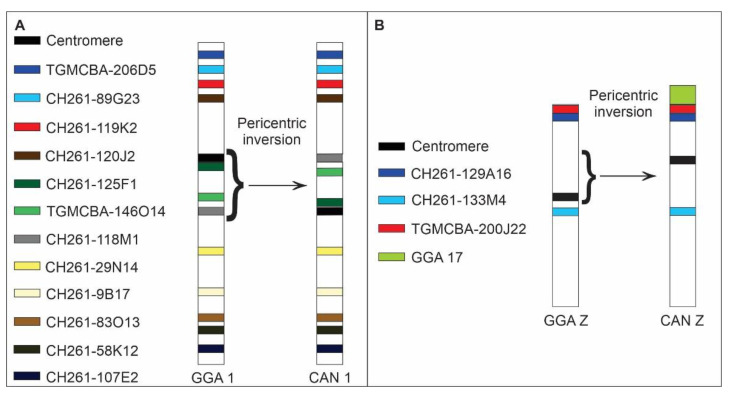
Schematic representation of chromosome localization of chicken (CH261) and zebra finch (TGMCBA) BACs homologous to chicken chromosome 1 (GGA 1) (**A**) and Z (GGAZ) (**B**) in *Crotophaga ani* (CAN). The BACs used and centromeres are indicated by the colours. The braces indicate the intrachromosomal rearrangement detected. A pericentric inversion occurred in chromosome 1 of *C*. *ani* (CAN 1) inverting the position of the centromere, CH261-125F1, TGMCBA-146O14, and CH261-118M1. A pericentric inversion also occurred in chromosome Z of *C*. *ani* (CAN Z).

Comparative chromosome mapping of BAC clones in *C*. *ani* revealed identical results in both individuals. Considering the interchromosomal rearrangements, a total of six fissions of macrochromosomes and nine fusions involving macrochromosomes and microchromosomes were detected when compared with chicken (Figure 1). Chicken chromosome 4 was split into four different pairs (CAN6p, CAN7, CAN9p, and CAN12p), chromosome 5 in three pairs (CAN4p, CAN5p, and CAN14), and chromosomes 6 and 8 are split into two pairs each (CAN4q and CAN11q, CAN8q, and CAN15, respectively). The following fusions were detected: GGA6/5 (CAN4), GGA15/5 (CAN5), GGA7/GGA4q (CAN6), GGA8/11 (CAN8), GGA12/GGA4p (CAN9), GGA10/25 (CAN10), GGA6/14 (CAN11), GGA13/GGA4q (CAN12), and GGAZ/17 (CANZ). The most unusual rearrangement detected was the Robertsonian translocation (i.e., centric fusions) between the Z and the chicken microchromosome 17. Among the BAC clones from chicken microchromosome 17 used, one of them, the CH261-42P16 produced signals in the CAN Z and CAN W, while the other BAC, the TGMCBA-375I5 produced signals only on the CAN Z. Representative FISH images are shown in Figure 3 and Figure 4. The chromosome mapping of BACs from chicken chromosome 1 and Z is shown in Figure 5, while the chromosome mapping of BACs from chicken chromosome 2-5 is shown in Figures S1–S4. Besides, four intrachromosomal rearrangements were found in the first five macrochromosomes pairs of *C*. *ani* (Figure 5 and Figures S1–S4).

## 4. Discussion

Since the first reports, cytogenetic studies on cuckoos have revealed karyotype variation both in chromosomal number and morphology, with 2n ranging from 64 to 90 [28,29,30]. This indicates that interchromosomal rearrangements, such as fusion, fission, and intrachromosomal rearrangements, such as pericentric inversion and centromere repositions, have played an important role in the chromosome evolution of these species. Here, analysing the karyotype of *C*. *ani* (2n = 74), we demonstrated an extensive chromosome reorganization involving fissions of macrochromosomes, fusions among macrochromosomes and microchromosomes, pericentric inversion, and centromere repositions in some macrochromosomes when compared with a chicken. These results have provided new insights into the karyotype relationships and genome evolution of cuckoos and detected an unusual Z-autosome Robertsonian translocation.

Waldrigues and Ferrari [28] previously reported the karyotype of *C*. *ani* with 2n = 70, after analysing nine specimens, however, the individuals here investigated appeared to have 2n = 74, with two additional pairs of microchromosomes. Nevertheless, the macrochromosomes morphology of *C*. *ani* found here agrees with Waldrigues and Ferrari [28], indicating that the difference in the 2n found was due to an inaccurate count of microchromosomes, a difficulty usually associated with the first attempts of analyses of avian karyotypes. 

The distribution of 18S rDNA clusters in cuckoos showed different evolutionary trajectories. Hence, *C*. *ani*, *Coccyzus melacoryphus*, *P*. *cayana*, and *G*. *guira* had these clusters in a single pair of chromosomes. However, in *P*. *cayana* and *G*. *guira* [36], this single pair is a macrochromosome, not homologous to the one observed in *C*. *ani* and *Coccyzus melacoryphus*, which in turn bears 18S rDNA sites in two microchromosomes. A single pair of microchromosomes with 18S rDNA clusters is considered as an ancestral feature, since it was observed in most of the avian species, including in ratites (ancestral avian clade) [3,37]. Therefore, different chromosome fusions or translocations events occurred in *P*. *cayana* and *G*. *guira*, while the ancestral state was conserved in *C*. *ani* and *C*. *melacoryphus*. This suggests that rDNA clusters may be important in the avian karyotype evolution as hotspots to chromosomal rearrangements.

Although SSR present distinct patterns of distributions among birds, these sequences tend to preferentially accumulate in the W and microchromosomes, usually associated with constitutive heterochromatin [8,9,38,39]. Similarly, in *C*. *ani* we found the SSRs to be amplified in several pairs of microchromosomes and in the W chromosome, however, it was not associated with constitutive heterochromatin, except in the W chromosome. In fact, the C-banding results indicated a low percentage of constitutive heterochromatin, restricted to a pericentric block in the W chromosome of *C*. *ani*. However, we also found SSRs to be accumulated in the Z chromosome, a rare fact among birds, although they have been observed in Piciformes species, which have a peculiar Z chromosome as the biggest chromosome in their karyotype [5,6]. Altogether, these facts highlight the role of these sequences in the sex chromosome differentiation in birds, where different species-specific features were revealed.

Our results with BACs probes demonstrated the occurrence of six fissions and nine fusions in *C*. *ani* when compared to *G*. *gallus*. Although several fissions occurred, extensive fusion events contributed to decreasing the 2n to 74, when compared to chicken (2n = 78). The comparison of our results with the data of *G*. *guira* from dos Santos et al. [30] suggests a very similar karyotype between these species, with several chromosomal rearrangements shared between both species (Table 1). On the other hand, no chromosomal rearrangements were shared between these species and *P*. *cayana*. This fact highlights the high similarity among the species within Crotophaginae subfamily while pointing to high karyotype diversity in Cuculidae.

The results of whole chromosome paints from macrochromosomes of chicken and white hawk revealed some gaps in the macrochromosomes of *G*. *guira* and *P*. *cayana*, indicating that chromosomal evolution of these species involved rearrangements between macro and microchromosomes [30]. Here, we were able to confirm the occurrence of fusions involving microchromosomes corresponding to GGA 11-15, 17, and 25 in *C*. *ani*. Microchromosome fusions have been only found in Falconiformes and Psittaciformes [22,23,24,25], while in the other eight avian orders no evidence of fusions involving these elements has been observed [25].

Among the fusions detected in *C*. *ani*, the most unexpected was a Z-autosome Robertsonian translocation. This type of rearrangement is rare in birds and has been described only in some individuals of *G*. *gallus* [13,14] and in Sylvioidea species (Passeriformes) [15,16,17,18]. While in *G*. *gallus* the rearrangements were identified just in some individuals and represent chromosome abnormalities, in Sylvioidea species they appear to be fixed. Interestingly, the Z chromosome of the dragon lizard (*Pogona vitticeps*) has homology with the chicken chromosome 17, besides the chromosome 23 [40]. This suggests that the chicken chromosome 17 have been recruited as sex chromosomes recurrently among amniotes, and may have some selective pressure to this translocation that had occurred in *C*. *ani*. Sigeman et al. [18] suggested that Sylvioidea species might be especially prone to the emergence and fixation of Z-autosome Robertsonian translocation, which may also be the case of cuckoo species. Hence, future studies are necessary to investigate if the Z-autosome Robertsonian translocation found here is unique of *C*. *ani*, or if it is shared among the other cuckoos species, especially the three species from the same subfamily: *C*. *major*, *C*. *sulcirostris*, and *G*. *guira* [27].

Although both BAC clones from chicken microchromosome 17 tested in our study produced signals on the Z chromosome of *C*. *ani*, one of them, the BAC CH261-4216 produced additional signals in the W chromosome. This result suggests that the BAC CH261-4216 had similar DNA sequence region with the *C*. *ani* W chromosome, probably corresponding to repetitive DNAs. Corroborating this hypothesis, the SSRs (GA)_15_ and the BAC CH261-133M4 (GGA Z) also produced signals in the same region of the BAC CH261-4216 on the *C*. *ani* W chromosome.

The consequence of sex–autosome translocations in birds remains unknown, probably due to the few cases of this type of rearrangement described in the literature. In mammals, the sex–autosome fusions have been associated with deleterious effects in humans and mice due to the inactivation of the autosomal segment translocated to the sex chromosome [41,42,43]. However, several authors have proposed that repetitive sequences-rich regions, such as heterochromatin blocks, intercalated between the ancestral chromosome fused to the sex chromosomes can act as a barrier to the progression of meiotic sex chromosome inactivation to the autosomal segment in species with these rearrangements [10,44,45]. On the other hand, in the common shrew (*Sorex araneus*), the translocated autosomal element onto the X chromosome did not affect the behaviour of the true sex chromosome regions in meiosis and did not affect the process of chromatin transformation at prophase I [46].

In birds, there is a lack of global inactivation mechanisms [47,48,49], therefore a different mechanism may have evolved to solve the dosage compensation in species with sex–autosome fusions, such as in *C*. *ani*. Comparing the ratio of expression of Z-linked genes by microarray and transcriptome data showed that the expression was significantly higher for Z genes in males than in females in zebra finch and chicken [50,51,52]. Nevertheless, partial upregulation restricted to the heterogametic sex (ZW, females) on the avian Z chromosome explains the partially sex-biased Z expression and a lack of global inactivation mechanisms [52]. Similarly, we suggest that the upregulation of the autosome chromosome region fused to the Z in females of *C*. *ani* is the mechanism to balance the gene expression between males and females. This mechanism is crucial to the successful fixation of the Robertsonian translocation between the microchromosome 17 and the Z chromosome in *C*. *ani*.

## 5. Conclusions

Our cytogenetic analysis demonstrates a remarkable chromosomal reorganization in *C*. *ani*, involving both inter and intrachromosomal rearrangements. Although *C*. *ani* has undergone six fissions, extensive chromosome fusions decreased its 2n to 74. Several microchromosomes were involved in fusion events, indicating that microchromosomes fusions are not exclusive to Falconiformes and Psittaciformes. Among the fusions detected, the most surprising was a Z-autosome translocation, a rare event in birds. Considering the chromosomal peculiarities detected in *C*. *ani*, it is anticipated that our analysis will encourage future studies using cuckoos as a model.

## Figures and Tables

**Table 1:** Patterns of interchromosomal rearrangements revealed in the karyotype of cuckoos species.

Chicken Chromosomes	Equivalent Guira Cuckoo, *Guira Guira* [30]	Equivalent Squirrel Cuckoo, *Piaya Cayana* [30]	Equivalent Smooth Billed Ani, *Crotophaga Ani* (Present Study)
1	1	1 and 6	1
2	2	2, 13 and 15	2
3	3	5 and 10	3
4q	4p and 6	3	6p, 7 and 12p
4p	9p	14	9p
5	5q, 7p and 8p	4	4p, 5p and 14
6	8q and 12q	8	4q and 11q
7	4q and 10	7	6q
8	9q	9	8q
9	11	11	13
10	5p	12	10q
11	-	-	8p
12	-	-	9q
13	-	-	12q
14	-	-	11p
15	-	-	5q
16	-	-	No data
17	-	-	Z
18	-	-	17
19	-	-	18
20	-	-	19
21	-	-	20
22	-	-	21
23	-	-	22
24	-	-	23
25	-	-	10p
26	-	-	24
27	-	-	25
28	-	-	26

## Supplementary Materials

The following are available online at https://www.mdpi.com/2073-4409/10/1/4/s1, Figure S1: Schematic representation of chromosome localization of chicken (CH261) and zebra finch (TGMCBA) BACs homologous to chicken chromosome 2 (GGA2) used in *Crotophaga ani* (CAN). Figure S2: Schematic representation of chromosome localization of chicken (CH261) and zebra finch (TGMCBA) BACs homologous to chicken chromosome 3 (GGA3) used in *Crotophaga ani* (CAN). Figure S3: Schematic representation of chromosome localization of chicken (CH261) and zebra finch (TGMCBA) BACs homologous to chicken chromosome 4 (GGA4) used in *Crotophaga ani* (CAN). Figure S4: Schematic representation of chromosome localization of chicken (CH261) and zebra finch (TGMCBA) BACs homologous to chicken chromosome 5 (GGA 5) used in *Crotophaga ani* (CAN). Table S1: List of BAC applied to *Crotophaga ani* (CAN).
