# Supplementary material for: Chromosomal Analysis in Crotophaga ani (Aves, Cuculiformes) Reveals Extensive Genomic Reorganization and an Unusual Z-Autosome Robertsonian Translocation
Source: Cells. 2020 Dec 22;10(1):4. doi: 10.3390/cells10010004 (PMC7822047; doi:10.3390/cells10010004)
Supplement: Supplementary file 1 [file cells-10-00004-s001.pdf]

Chromosomal analysis in *Crotophaga ani* (Aves, Cuculiformes) reveals extensive genomic reorganization and an unusual Z-autosome Robertsonian translocation

Rafael Kretschmer[1,2,*], Ricardo José Gunski[3], Analía del Valle Garnero[3], Thales Renato Ochotorena de Freitas[2], Gustavo Akira Toma[4], Marcelo de Bello Cioffi[4], Edivaldo Herculano Corrêa de Oliveira[5,6], Rebecca E. O'Connor[1], Darren K. Griffin[1]

School of Biosciences, University of Kent, CT2 7NJ Canterbury, UK; rebeckyoc@gmail.com (R.E.O.); d.k.griffin@kent.ac.uk (D.K.G.)

[2] Laboratório de Citogenética e Evolução, Departamento de Genética, Instituto de Biociências, Universidade Federal do Rio Grande do Sul, Porto Alegre 91509-900, Rio Grande do Sul, Brazil; thales.freitas@ufrgs.br (T.R.O.d.F.)

[3] Laboratório de Diversidade Genética Animal, Universidade Federal do Pampa, São Gabriel 97300-162, Rio Grande do Sul, Brazil; ricardogunski@unipampa.edu.br (R.J.G.); analiagarnero@unipampa.edu.br (A.d.V.G.)

[4] Laboratório de Citogenética de Peixes, Departamento de Genética e Evolução, Centro de Ciências Biológicas e da Saúde, Universidade Federal de São Carlos, São Carlos 13565-905, São Paulo, Brazil; gustavo_toma@hotmail.com (G.A.T.); mbcioffi@ufscar.br (M.d.B.C.)

[5] Laboratório de Cultura de Tecidos e Citogenética, SAMAM, Instituto Evandro Chagas, Ananindeua 67030-000, Pará, Brazil; ehco@ufpa.br (E.H.C.d.O.)

[6] Instituto de Ciências Exatas e Naturais, Universidade Federal do Pará, Belém 66075-110, Pará, Brazil;

* Correspondence: rafa.kretschmer@hotmail.com

SUPPLEMENTARY INFORMATION

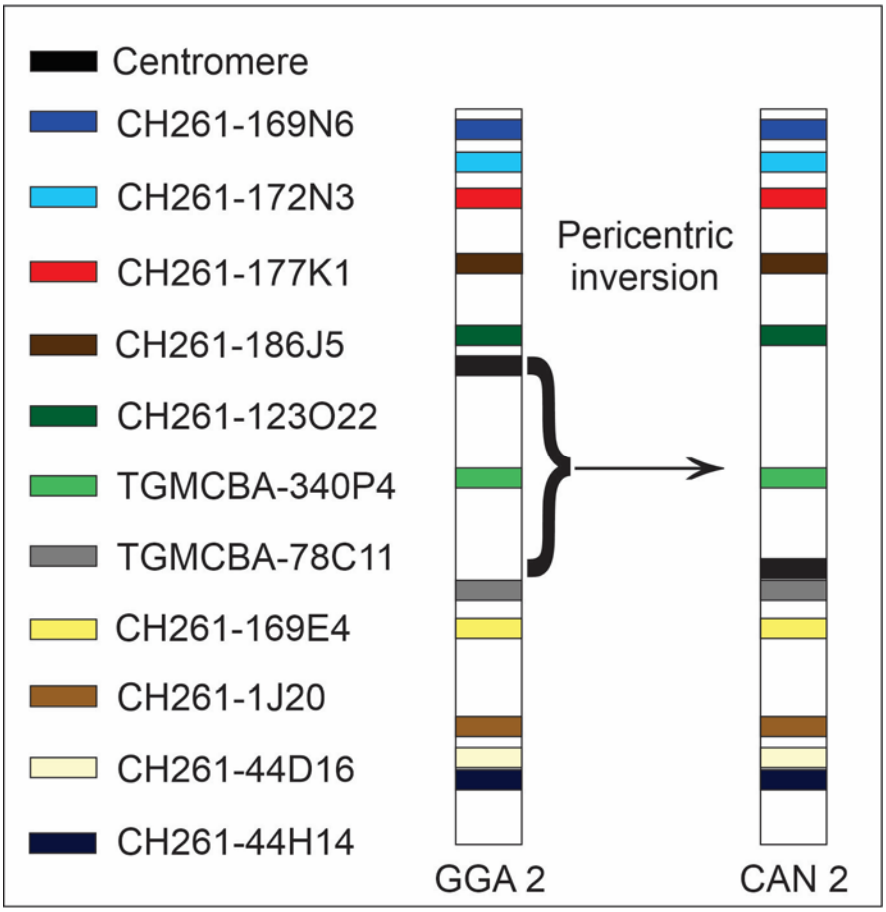

Figure S1: Schematic representation of chromosome localization of chicken (CH261) and zebra finch (TGMCBA) BACs homologous to chicken chromosome 2 (GGA2) used in *Crotophaga ani* (CAN). The BACs used and centromeres are indicated by the colors. The braces indicate the chromosomal rearrangements detected. A centromere reposition or pericentric inversion originated the chromosome 2 of *C. ani* (CAN2).

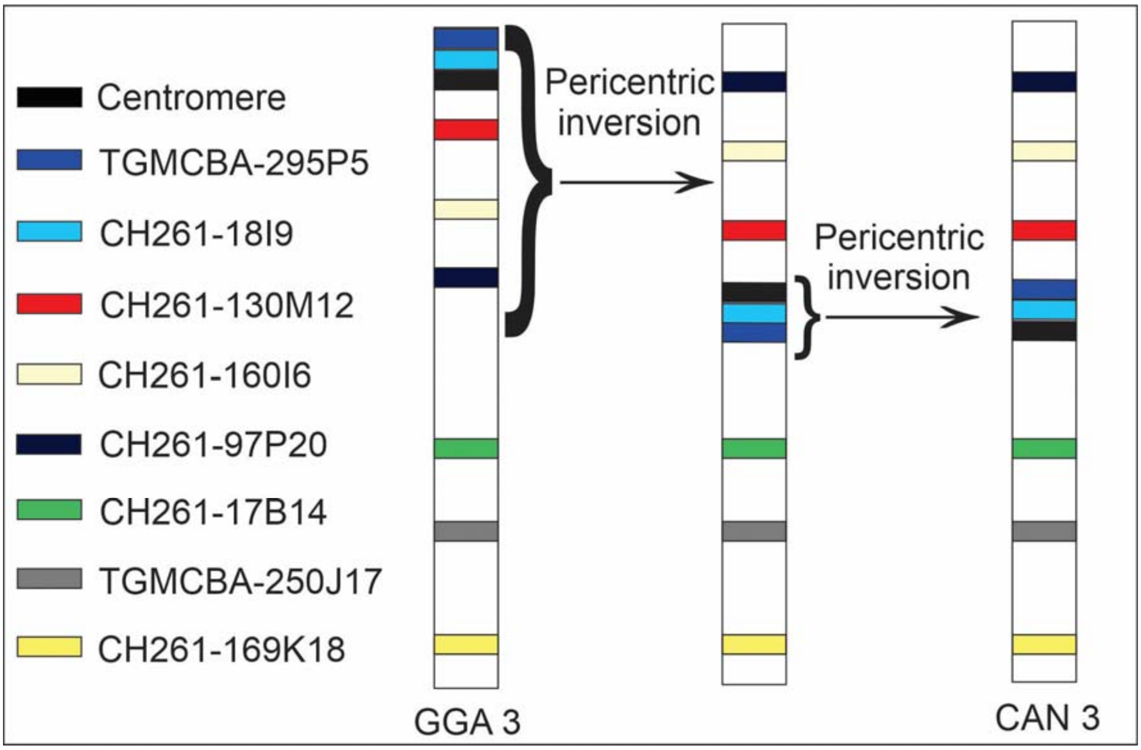

Figure S2: Schematic representation of chromosome localization of chicken (CH261) and zebra finch (TGMCBA) BACs homologous to chicken chromosome 3 (GGA3) used in *Crotophaga ani* (CAN). The BACs used and centromeres are indicated by the colors. The braces indicate the chromosomal rearrangements detected. Two pericentric inversions occurred in the chromosome 3 of *C. ani* (CAN3).

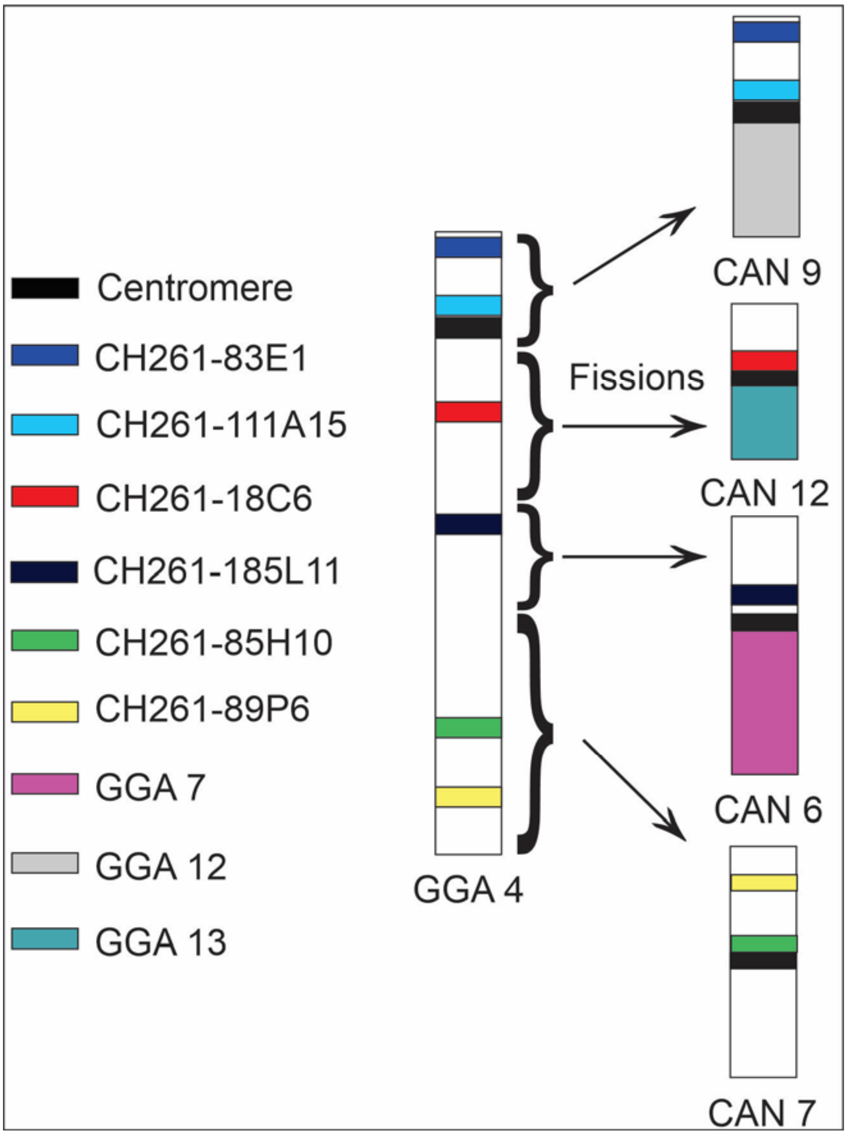

Figure S3: Schematic representation of chromosome localization of chicken (CH261) and zebra finch (TGMCBA) BACs homologous to chicken chromosome 4 (GGA4) used in *Crotophaga ani* (CAN). The BACs used and centromeres are indicated by the colors. The braces indicate the chromosomal rearrangements detected. The GGA4p was preserved as a separated chromosome in *C. ani* (CAN9), as in the ancestral bird karyotype. Two fissions events have occurred in ancestral chromosome GGA4q in *C. ani*, originating the chromosome CAN6, 7 and 12.

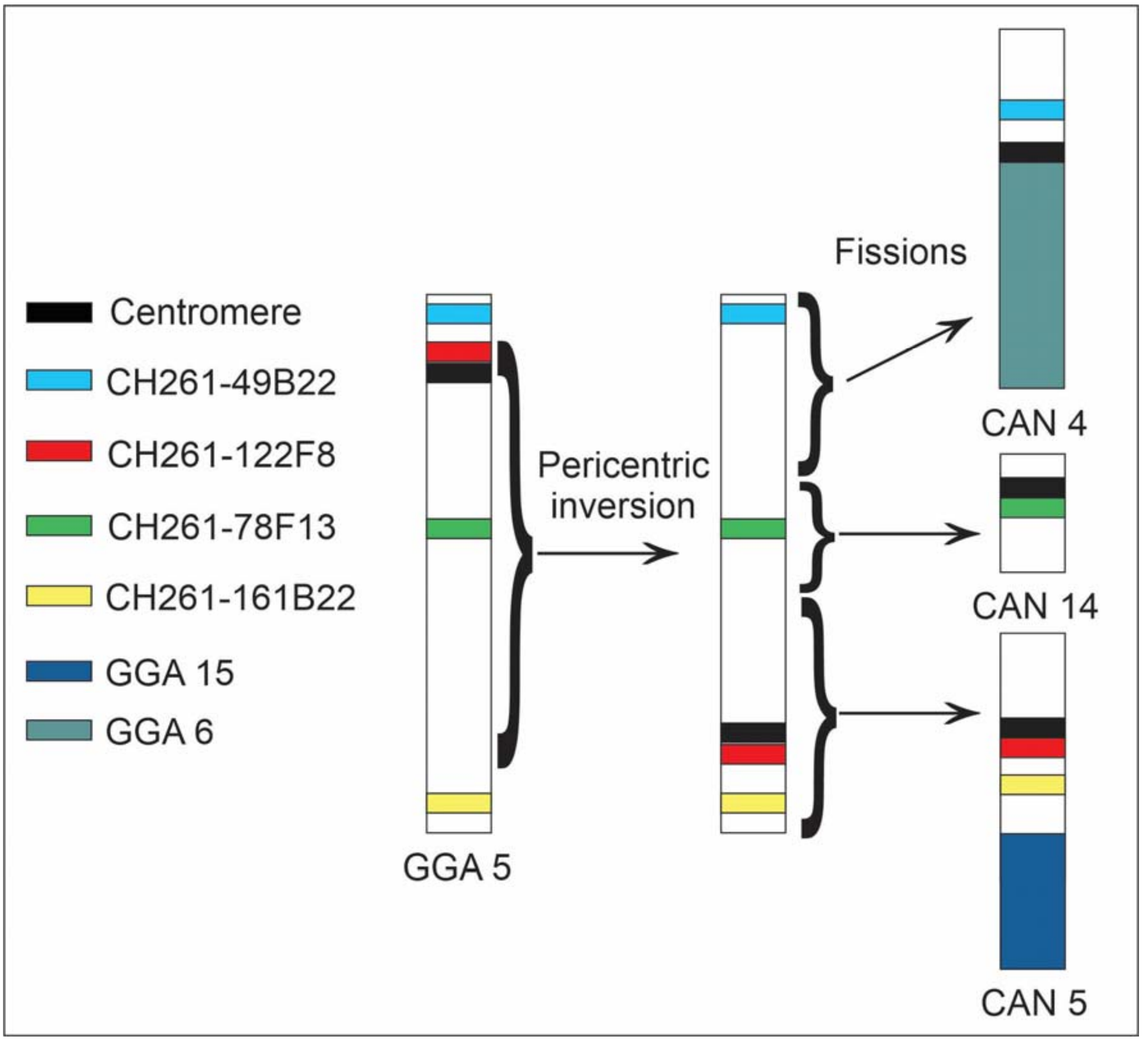

Figure S4: Schematic representation of chromosome localization of chicken (CH261) and zebra finch (TGMCBA) BACs homologous to chicken chromosome 5 (GGA 5) used in *Crotophaga ani* (CAN). The BACs used and centromeres are indicated by the colors. The braces indicate the chromosomal rearrangements detected. One pericentric inversion and two fissions occurred in the GGA 5 of *C. ani*. Additionally, a segment from GGA 5 fused to a segment from GGA 6 (CAN 4) and other segment from GGA 5 fused to GGA 15 (CAN 5).

Table S1: List of BAC applied to *Crotophaga ani* (CAN).

BAC ID	GGA Chr	CAN Chr
TGMCBA-206D5	1	1
CH261-89G23	1	1
CH261-119K2	1	1
CH261-120J2	1	1
CH261-125F1	1	1

TGMCBA-146O14	1	1
CH261-118M1	1	1
CH261-29N14	1	1
CH261-9B17	1	1
CH261-83O13	1	1
CH261-107E2	1	1
CH261-58K12	1	1
CH261-169N6	2	2
CH261-172N3	2	2
CH261-177K1	2	2
CH261-186J5	2	2
CH261-123O22	2	2
TGMCBA-340P4	2	2
TGMCBA-78C11	2	2
CH261-169E4	2	2
CH261-1J20	2	2
CH261-44H16	2	2
CH261-44H14	2	2
TGMCBA-295P5	3	3
CH261-18I9	3	3
CH261-130M12	3	3
CH261-160I6	3	3
CH261-97P20	3	3
CH261-17B14	3	3
TGMCBA-250J17	3	3
CH261-169K18	3	3
CH261-83E1	4	9
CH261-111A15	4	9
CH261-18C6	4	12p
CH261-185L11	4	7
CH261-85H10	4	6
CH261-89P6	4	6
CH261-49B22	5	4
CH261-78F13	5	14
CH261-122F8	5	5
CH261-161B22	5	5
TGMCBA-382J4	6	4
CH261-49F3	6	11q
CH261-56K7	7	6
CH261-180H18	7	6
CH261-107D8	8	8
TGMCBA-252A4	8	15
CH261-183N19	9	13

CH261-187M16	9	13
CH261-115G24	10	10
CH261-71G18	10	10
CH261-154H1	11	8
CH261-121N21	11	8
CH261-60P3	12	9q
CH261-4M5	12	9q
TGMCBA-321B13	13	12q
CH261-115I12	13	12q
CH261-122H14	14	11p
CH261-69D20	14	11p
CH261-90P23	15	5q
TGMCBA-266G23	15	5q
TGMCBA-375I5	17	Zp
CH261-42P16	17	Zp
CH261-60N6	18	17
CH261-72B18	18	17
CH261-10F1	19	18
CH261-50H12	19	18
TGMCBA-250E3	20	19
TGMCBA-341F20	20	19
CH261-83I20	21	20
CH261-122K8	21	20
CH261-40J9	22	21
CH261-18G17	22	21
CH261-191G17	23	22
CH261-90K11	23	22
CH261-103F4	24	23
CH261-65O4	24	23
CH261-59C21	25	10
CH261-127K7	25	10
CH261-186M13	26	24
CH261-170L23	26	24
CH261-66M16	27	25
CH261-28L10	27	25
CH261-72A10	28	26
CH261-64A15	28	26
CH261-129A16	Z	Z
CH261-133M4	Z	Z
TGMCBA-200J22	Z	Z
CH261-94E12	W	W
